# Horse-riding hazards: an observational cohort study mapping equestrian related injuries at a Scandinavian trauma centre

**DOI:** 10.1186/s13102-023-00646-y

**Published:** 2023-03-28

**Authors:** Emilie Franzén Lindgren, Folke Hammarqvist, Rebecka Ahl Hulme

**Affiliations:** 1 Medical School, Karolinska Institute, Stockholm, Sweden; 2 Division of Surgery, Department of Clinical Science, Intervention and Technology, Karolinska Institute, Stockholm, Sweden; 3 Division of Trauma and Emergency Surgery, Department of Surgery, Karolinska University Hospital, Stockholm, Sweden

**Keywords:** Equestrian trauma, Horse related injuries, Horse riding

## Abstract

**Introduction:**

Horse-riding is practiced on a regular basis by 500,000 people in Sweden. It is reputed to be one of the most dangerous sports. On average, there were 1756 acute injuries and three fatalities each year between 1997 and 2014 in Sweden related to horses. The primary aim of this study was to outline the injury spectrum related to equestrian activities cared for at a large Swedish trauma centre. The secondary aim was to identify trends in clinical outcomes and to investigate the association between age and such outcomes.

**Material and methods:**

The electronic medical records system at Karolinska University Hospital was queried for patients cared for due to equestrian related trauma between July 2010 and July 2020. Complementary data were gathered using the hospital’s Trauma Registry. No exclusion criteria were applied. Descriptive statistics were used to outline the injury spectrum. Age was split into four categories which were compared using the Kruskal–Wallis H test or the Chi-squared test. Logistic regression was used to analyse correlations between age and outcomes.

**Results:**

A total of 3036 patients were included with 3325 injuries identified as equestrian related. The hospital admission rate was 24.9%. The cohort had one death. Regression analysis showed significant associations between decreasing risk of upper extremity injury (p < 0.001), increasing risk of vertebral fractures (p = 0.001) and increasing risk of thoracic injury (p < 0.001) with increasing age.

**Conclusions:**

Equestrian activities are not without risks. The morbidity is high, and injuries are taken seriously by the medical profession, reflected by the high admission rate. There are age-related variations in the injury spectrum. Older age appears to predispose to vertebral fractures and thoracic injuries. Other factors than age appear more important in determining the need for surgery or admission to ICU.

## Introduction

There are nearly 390 horses per 10,000 inhabitants in Sweden. A report from 2015 by the World Horse Welfare and the Eurogroup for Animals places Sweden in the top five European member states of horses per capita, with 23.7 horses per 1000 people. The only European countries with a higher prevalence are Belgium, Romania and Ireland [1]. Equestrian activities provide approximately 38,000 full-time jobs and around 500,000 people in Sweden practices horse riding on a regular basis [2]. Horse riding has the reputation of being a dangerous sport. Between 1997 and 2014, there were 1756 injuries and three fatalities yearly in Sweden due to equestrian activities [3, 4]. This equates to an injury rate of approximately 18 cases per 100,000 population. The conception of horse riding as a dangerous activity is thought to be derived from eventing. Eventing consists of dressage, show jumping and cross country. Cross-country, in particular, is faced with many risks. Obstacles on the course includes waters, up jumps, steeps, solid fences and long hauls in between. The speed needs to be high, and the length of the strides long enough for the horse to obtain adequate power to get over the obstacles. This aggravates the ride as the possibility to plan or correct each jump off is reduced. Most obstacles during a cross country course are solid. Consequently, if the rider fails to make a correct jump off, the fence will not fall, but rather cause the horse to somersault over it. The horse will make an abrupt halt while the rider is plunged forward with continued momentum. Additionally, the rider will most likely end up underneath the somersaulting horse as it lands. This is called a rotational fall and is the most common cause of fatalities during eventing [5].

Safety rules and regulations of the equestrian community in Sweden is set by the Swedish Equestrian Federation (SEF). A helmet of a certain certification must be worn when participating in any activities connected to the SEF. The use of helmets is more frequent nowadays and have reduced the incidence of head injuries. Nevertheless, injuries to the head are still the most frequent and most lethal injuries [6]. Riders under eighteen are required to wear a safety vest when competing in show jumping. At the cross country course, riders of all ages must wear a safety vest. The use of safety vests is well established, especially amongst younger riders. However, the effectiveness of the vest outside of the cross country course still lacks sound scientific evidence [7]. A recent study suggests that a certain type of safety vest (an inflatable vest) could even constitute a risk for the rider rather than protection [8]. Speculations on why the effectiveness of the vests differs from cross country to other disciplines suggest that the answer lies in the nature of the activities [7].

Eventing is a relatively small part of the equestrian community and can therefore not explain all injuries related to horses. Only 1.7% of all competitions in Sweden during 2019 were eventing [9], this implies that most riders are dedicated to other disciplines such as show jumping and dressage. With 450 riding schools in Sweden there are 5 million hours of riding each year at the clubs alone. Despite the vast number of hours spent in the saddle, severe injuries are uncommon when riding with a licensed trainer or at a riding school [10]. Horse riding has a significant impact on the public health system in Sweden. Each injury sustained during equestrian activities is estimated to cost around 1800 euro, equivalent to 3.2 million euro per year [4]. The insurance company Folksam covers all members of the SEF during any activities related to the federation. In 2017, they had 907 insurance claims regarding horse related injuries. This equates to 0.6% of all SEF members at that time. There was a total of 1132 injuries in these 907 incidents and the most common areas to be injured were head and neck (36.4% of cases). Soft tissue damage was the most common injury (39%) and in second place were fractures and dislocations (36.7%) [3]. However, if the rider did not make an insurance claim they will not show up in these statistics. The above figures, therefore, most likely underestimate the true numbers. The Swedish Civil Contingencies Agency published a report using national data from 2005 to 2007 that outline the healthcare burden, in terms of days spent in hospital, as a consequence of activity related sustained injuries. The report showed that equestrian related activities make up 33.3% of all days spent in hospital. This was 2.7 times the number of days in hospital caused by motorsport injuries, 1.9 times that of football injuries and 2.3 times that of downhill skiing. In addition, equestrian related injuries lead to, on average, 0.87 days in hospital per injury, which is the highest average of any of the sports listed by the report [11]. The same report estimated a yearly economic burden of 1.3 billion SEK ($\approx$ 126 million USD) for hospital costs from physical activity related injuries. Of this, equestrian related activities accounted for approximately 6.5%. This number does not, however, account for costs incurred from rehabilitation, permanent physical disabilities and any loss of income.

It is a general assumption that horse riding comes with risks and there are varying suggestions on how to minimise these. Some suggest that if riders have more understanding about horses and their behaviour it could reduce the risk, advocating an approach called Natural Horsemanship [12]. Others suggest that the best thing would be to diminish the severity of the injuries by identifying the best safety equipment [13, 14]. One can argue whether focus should be on preventing accidents from happening, or on protection wear for when they occur. Whichever argument you choose, there is need for improvements regarding the safety of horse riders. To learn how to avoid the risks and how best to treat injuries when they do occur, they first need to be identified. Presently, scientific evidence is sparse in this area. To learn more about the risks of equestrian activities, the primary aim of this study was to map equestrian related injuries treated at a large Swedish trauma centre. The secondary aim was to describe clinical outcomes and specifically explore any association between age and such outcomes.

## Material & methods

### Patient selection and data collection

This retrospective observational cohort study was granted ethical approval from the Swedish Ethical Review Authority (“Etikprövningsmyndigheten”) (ref. 2021–03330) and was carried out in accordance with the standards laid down in the Declaration of Helsinki. All patients suffering equestrian related injuries between 31 July 2010 and 31 July 2020 at Karolinska University Hospital in Stockholm, Sweden were included. An equestrian related injury was defined as any event involving a horse that led to the patient seeking medical attention at the hospital. Equestrian related events and injury diagnoses were identified using ICD-10 codes. Karolinska University Hospital is the largest trauma hospital in Sweden with a catchment population of over 2.5 million. Patients of all ages were included. No exclusion criteria were applied. Data variables were collected from electronic medical records and from the Karolinska Trauma Registry. Variables collected included sex, age, Glasgow Coma Scale (GCS) upon arrival, Charlson Comorbidity Index (CCI), trauma team activation level (TAL), mechanism of injury (MOI), admission findings, hospital length of stay (HLOS), intensive care length of stay (ICU LOS), in-hospital mortality, surgery, number of injured body areas (NOI), Injury Severity Score (ISS), type of injury and systolic blood pressure (SBP) on arrival. Injuries, coded using ICD-10, were sorted according to type of injury and body regions: traumatic brain injury (TBI), skull/scalp, face, neck, vertebral column, spine, abdomen, thorax, pelvis, urogenital tract, upper extremity, and lower extremity. TBI was defined as any form of intracranial injury including concussions. Upper extremity injury was defined as any injury from the shoulder to the fingertips. Lower extremity injury was defined as any injury from the hip to the toes. Injury to the urogenital tract was limited to injuries to the genitals, urethra, bladder, and ureters. Traumatic kidney injury was included under the subcategory of abdominal injury.

### Statistics

Collected data were analysed using the statistical programme SPSS and statistical significance was considered at a p-value of < 0.05. Descriptive statistics were conducted. On all continuous variables normality tests were conducted using the Shapiro–Wilk and the Kolmogorov Smirnov tests when appropriate. Patients were first analysed as a full cohort and later subdivided for subgroup analyses. The first subdivision separated patients who were admitted to hospital from those who were sent home from the Emergency Department (ED). The group of admitted patients were further subdivided into four groups based on the patients’ age at the time of the injury. The age limits of these groups were chosen arbitrarily but based on consideration to what is age-typical for equestrian activities and age limits that are traditionally relevant for traumatic injury: Group 1 consisted of 1–10-year-olds i.e., riders that are likely to have less experience and therefore not the same balance of older and more experienced riders, Group 2 was made up of 11–17 year-olds i.e., riders who have more experience due to the increasing age. The riding is expected to involve more risk taking compared to younger riders, Group 3 reflected the adult group with the age interval 18–54 years, Group 4 consisted of patients aged 55 years and older. In trauma related research this age group of elderly patients is associated with worse outcomes following traumatic injury.

The four age groups were compared to identify possible age differences. The Kruskal–Wallis H test was performed on continuous and ordinal variables. The Chi-Square test was used for categorical variables. Outcomes included in the analyses were ICU, need for surgery and hospital length of stay. Analysed injuries were limited to the five most frequent types. Patient variables that from the Kruskal–Wallis H and Chi-Square tests appeared to have a possible association with age (i.e., p value < 0.2) were included in subsequent multivariate logistic regression models. Five models were analysed to explore five dependent outcomes: ICU, surgery, extremity injury, vertebral fractures, and thoracic injuries. Analysed injuries were limited to the three types with the lowest p values from the Kruskal–Wallis H or Chi-Square tests.

## Results

### Demographics

**Fig. 1:**
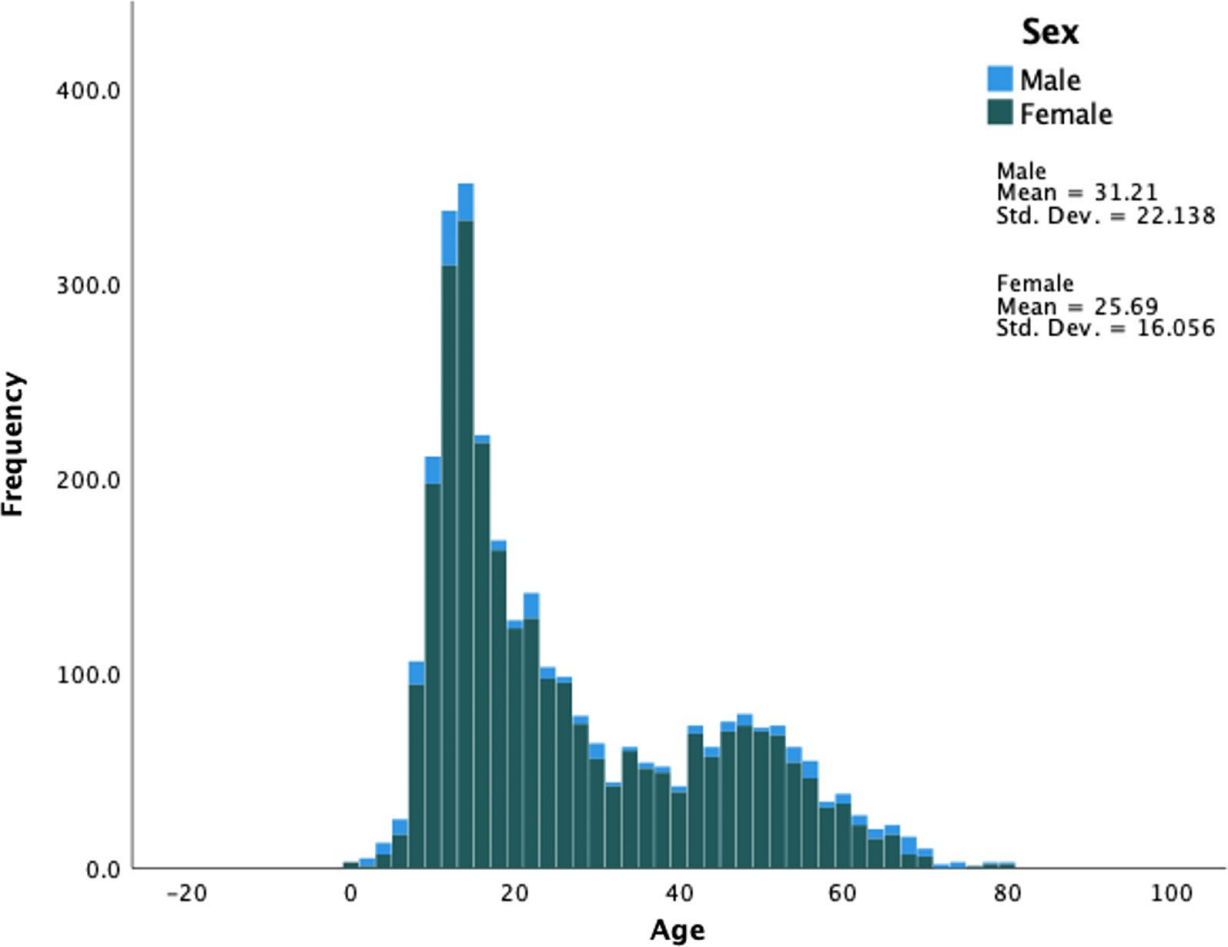
Distribution of patients and their sex at each age at the time of the injury

A total of 3036 patients with equestrian related injuries were identified. There was an overwhelming majority of female patients summing up to 92.2% (n = 2798) of the total cohort. The median [lower quartile, upper quartile] age was 20 [13, 39] years and the age distribution was wide with patients ranging from < 1 to 80 years old. The incidence of injuries was higher for children (see Fig. 1).

In the total cohort of 3036 patients, 3325 equestrian related injuries were identified. Table 1 outlines the distribution of sustained injuries in the total cohort. Injury to the upper extremity was the most common injury (25.6%, n = 851), followed by lower extremity injury (16.8%, n = 558) and traumatic brain injury (17.1%, n = 568). Of the total cohort of 3036 patients, 75.1% (n = 2279) were non-admissions. A total of 2334 injuries were registered for non-admissions while 186 patients obtained ICD-10 codes for pain in various body regions but without any registered physical injuries. Consequently, 8.2% of non-admissions did not sustain any visible internal or external injuries. Twenty-four-point nine percent (n = 757) of patients were admitted. This subgroup had a total of 991 registered injuries. Only 4.9% (n = 37) of them had no injuries and were admitted for observation, often due to an increased risk of bleeding as a consequence of anticoagulant therapy.

**Table 1:** Injury spectrum of the total cohort of 3036 patients with a total of 3325 registered injuries

Type of injury	N (%)*
*TBI*	568 (17.1)
SAH	18 (0.5)
SDH	11 (0.3)
EDH	1 (0.0)
Contusion	6 (0.2)
DAI	2 (0.1)
Concussion	496 (14.9)
UII	13 (0.4)
Multiple	21 (0.6)
*Scalp lacerations* *Skull fracture*	27 (0.8) 25 (0.8)
*Facial injury*	321 (9.7)
Skin wound	68 (2.0)
Soft tissue	16 (0.5)
Fracture	57 (1.7)
Injury to teeth	9 (0.3)
UIH	55 (1.7)
Superficial injury	106 (3.2)
Multiple	10 (0.3)
*Neck injury*	172 (5.2)
Neck fracture or distortion	110 (3.3)
Superficial injury	62 (1.7)
*Vertebral column injury*	344 (10.3)
Fracture	191 (5.7)
Distortion	11 (0.3)
Contusion	142 (4.3)
*Spinal cord injury*	9 (0.3)
*Abdominal injury*	92 (2.8)
Liver	14 (0.4)
Spleen	18 (0.5)
Kidney	8 (0.2)
Injury to abdominal wall	14 (0.4)
Intestine	2 (0.1)
Other bleeding	2 (0.1)
Other	27 (0.8)
Multiple	7 (0.2)
*Thoracic injury*	262 (7.9)
Rib fracture(s)	77 (2.3)
Pneumothorax	41 (1.2)
Lung contusion	18 (0.5)
Injury to thoracic wall	7 (0.2)
Fractured sternum	1 (0.0)
Distortion	1 (0.0)
Contusion	61 (1.8)
Superficial/Other	5 (0.2)
Multiple	51 (1.5)
*Pelvic fracture*	82 (2.5)
*Urogenital injury*	14 (0.4)
*Upper extremity injury*	851 (25.6)
Soft tissue/sprain/subluxation	234 (7.0)
Skin wound	28 (0.8)
Fracture	585 (17.6)
Multiple	4 (0.1)
*Lower extremity injury*	558 (16.8)
Soft tissue/sprain/subluxation	350 (10.5)
Skin wound	40 (1.2)
Fracture	165 (5.0)
Multiple	3 (0.1)

*TBI* traumatic brain injury, *SAH* subarachnoid haemorrhage, *SDH* subdural haemorrhage, *EDH* epidural haemorrhage, *DAI* diffuse axonal injury, *UII* unspecified intracranial injury, *UIH* unspecified injury to the head

*Percentage is in relation to the total number of injuries (n = 3325)

Patients admitted to hospital were further analysed. This subgroup remained dominated by the female sex (92.5%, n = 700) and had a low median age of 24 [13, 46] years. Only 9.2% (n = 70) required a level 1 TAL and the most common mechanism of injury was falling off the horse occurring in 84.3% (n = 638) (see Table 2).

**Table 2:** Demographics of patients admitted to hospital (n = 757)

Variable	
Age, median [Q1, Q3] (min, max)	24 [13, 46] (2, 80)
*Sex*	
Female, n (%)	700 (92.5)
Male, n (%)	57 (7.5)
GCS, median [Q1, Q3] (min, max)	15 [15] (3, 15)
CCI, median [Q1, Q3] (min, max)	0 [0, 1] (0, 9)
*Trauma team activation*	
Walking in by themselves, n (%)	259 (34.2)
Trauma level 1, n (%)	70 (9.2)
Trauma level 2, n (%)	360 (47.6)
Transferred from another hospital, n (%)	68 (9)
*Mechanism of injury*	
Fall, n (%)	638 (84.3)
Kicked, n (%)	12 (1.6)
Crushed, n (%)	14 (1.8)
Multiple, n (%)	93 (12.3)
*Number of sustained injuries*	
0, n (%)	37 (4.9)
1, n (%)	529 (69.9)
2, n (%)	130 (17.2)
3, n (%)	48 (6.3)
4, n (%)	8 (1.1)
5, n (%)	2 (0.3)
6, n (%)	2 (0.3)
7, n (%)	1 (0.1)

*GCS* Glasgow Coma Scale, *CCI* Charlson Comorbidity Index, *Q1* lower quartile, *Q3* upper quartile

Patients admitted to hospital demonstrated a similar injury pattern to the subgroup that were discharged from the ED; 32.9% (n = 249) sustained injury to an upper extremity, 24.8% (n = 188) sustained a TBI, and 18.0% (n = 136) suffered lower extremity injury. Fractures dominated as the most common type of injury to both upper and lower extremities (see Table 3).

**Table 3:** Injury spectrum of patients admitted to hospital (n = 757)

Area and type of injury	N (%)*
*TBI*	188 (24.8)
None	569 (75.2)
Concussion	138 (18.2)
SAH	18 (2.4)
SDH	10 (1.3)
EDH	2 (0.3)
Contusion	8 (1.1)
DAI	1 (0.1)
Multiple	11 (1.5)
*Skull fracture*	16 (2.1)
*Facial injury*	52 (6.9)
None	705 (93.1)
Skin wound	13 (1.7)
Soft tissue	9 (1.2)
Fracture	17 (2.2)
Injury to teeth	3 (0.4)
Multiple	10 (1.3)
*Neck fracture*	42 (5.5)
*Vertebral column fracture*	103 (13.6)
*Spinal cord injury*	8 (1.1)
*Thoracic injury*	103 (13.6)
None	654 (86.4)
Rib fracture(s)	24 (3.2)
Pneumothorax	16 (2.1)
Lung contusion	12 (1.6)
Multiple	51 (6.7)
*Abdominal injury*	44 (5.8)
None	713 (94.2)
Liver	11 (1.5)
Spleen	12 (1.6)
Kidney	7 (0.9)
Injury to the abdominal wall	1 (0.1)
Bowel	2 (0.3)
Other bleeding	2 (0.3)
Other	2 (0.3)
Multiple	7 (0.9)
*Pelvic fracture*	50 (6.6)
* Upper extremity injury*	249 (32.9)
None	508 (67.1)
Spraining/soft tissue/subluxation	38 (5.0)
Skin wound	12 (1.6)
Fracture	199 (26.3)
*Lower extremity injury*	136 (18.0)
None	621(82.0)
Spraining/soft tissue/subluxation	50 (6.6)
Skin wound	8 (1.1)
Fracture	78 (10.3)
*No injury recorded*	37 (4.9)

*TBI* traumatic brain injury, *SAH* subarachnoid haemorrhage, *SDH* subdural haemorrhage, *EDH* epidural haemorrhage, *DAI* diffuse axonal injury

*Percentage is in relation to the total number of patients (n = 757)

Surgery was required in 43.3% (n = 328) of patients admitted to hospital. Orthopaedic surgery was the most common type of surgery, occurring in 82% (n = 268) of surgical cases. Second most common surgery was neurosurgery carried out in 2.2% (n = 17) of patients. The need for intensive care was low at 4.8% (n = 36) (see Table 4). There was only one death in the total cohort. Mortality is, therefore, not the subject of further analysis.

**Table 4:** Clinical outcomes of patients admitted to hospital

Variable	N (%)
Intensive care admission	36 (4.8)
Surgery	328 (43.3)
*Type of surgery*	
Abdominal	2 (0.3)
Orthopaedic	268 (35.4)
Plastic/ENT	17 (2.2)
Thoracic	11 (1.5)
Endovascular	5 (0.7)
Neurosurgical	17 (2.2)
Urological/gynaecological	1 (0.1)
Multiple	7 (0.9)

	Median [Q1, Q3] (min, max)
HLOS	2 [1, 3] (0, 63)
ICU LOS*	1 [1, 2.5] (1, 49)

*HLOS* hospital length of stay, *ICU LOS* intensive care unit length of stay, *ENT* ear nose throat, *Q1* lower quartile, *Q3* upper quartile

*Days of ICU for the subgroup of patients who were admitted to the ICU

A comparison between age categories identified variables with possible associations with age (see Table 5). Variables with a p value of < 0.2 in the Kruskal–Wallis H or Chi-squared tests in Table 5 were used in subsequent regression analyses.

**Table 5:** Differences between age groups (Group 1: 0–10 years, Group 2: 11–17 years, Group 3: 18–54 years, Group 4: 55 years and over) for patients admitted to hospital

Variable	Group 1 n = 91	Group 2 n = 206	Group 3 n = 365	Group 4 n = 95	H	χ^2^	p
Sex (% female within each age category)	95.6	97.1	93.2	76.8		χ^2^(3) = 41.158	< 0.001
CCI (mean rank)	256.55	252.51	398.52	695.59	H (3) = 425.028		< 0.001
GCS (mean rank)	396.61	381.12	376.18	364.42	H (3) = 5.575		0.134
TAL (% within each age category)						χ^2^(3) = 75.290	< 0.001
Walking in	57.1	49.0	23.0	23.2			
TAL 1	0.0	7.8	10.7	15.8			
TAL 2	34.1	35.9	55.9	53.7			
Transfer	8.8	7.3	10.4	7.4			
MOI (% within each age category)						χ^2^(3) = 23.312	0.006
Fall	95.6	87.4	79.2	86.3			
Kicked	0.0	1.5	2.2	1.1			
Crushed	0.0	0.5	2.5	4.2			
Multiple	4.4	10.7	16.2	8.4			
NOI (mean rank)	322.97	332.86	396.90	463.94	H (3) = 48.871		< 0.001
HLOS (mean rank)	263.57	302.29	423.26	485.84	H (3) = 94.160		< 0.001
ICU LOS (mean rank)	365.07	371.89	382.76	393.32	H (3) = 8.109		0.044
ICU admission (% within each age category)	1.1	2.9	5.8	8.4		χ^2^(3) = 7.851	0.049
Surgery (% within each age category)	76.9	43.2	35.3	42.1		χ^2^(3) = 51.365	< 0.001
TBI* (% within each age category)	12.1	28.6	25.2	27.4		χ^2^(3) = 9.873	0.020
Vertebral fracture* (% within each age category)	2.2	5.3	19.5	20.0		χ^2^(3) = 35.966	< 0.001
Upper extremity injury* (% within each age category)	72.5	35.4	23.0	27.4		χ^2^(3) = 82.818	< 0.001
Lower extremity injury* (% within each age category)	8.8	21.8	20.0	10.5		χ^2^(3) = 11.893	0.008
Thoracic injury* (% within each age category)	2.2	4.4	16.4	33.7		χ^2^(3) = 60.099	< 0.001

*CCI* Charlson Comorbidity Index, *GCS* Glasgow Coma Scale, *TAL* trauma alert level, *MOI* mechanism of injury, *HLOS* hospital length of stay, *ICU LOS* ICU length of stay care, *ICU* intensive care unit, *NOI* number of injuries, *TBI* traumatic brain injury

*Percentages may add up to less or more than 100% for each age category due to some patients sustaining multiple injuries

While bivariate logistic regression analysis demonstrated a significant association between the need for intensive care and increasing age (odds ratio (OR) 1.03 95%CI 1.01–1.04, p = 0.004), the multivariate logistic regression model, adjusted for the patient variables sex, age, GCS on admission, CCI, trauma alert level, the mechanism of injury and whether the patient sustained multiple injuries of not, resulted in a loss of statistical significance (OR 1.05 95% CI 0.99–1.11, p = 0.092). A statistically significant inverse relationship was found between age and the need for surgery and was strengthened in the multivariate regression model (OR 0.98 95% CI 0.96–0.99, p = 0.022). Multivariate regression analysis demonstrated a significant relationship between age and injuries to the upper extremities, vertebral column and thorax. The risk of upper extremity injury showed a decrease with increasing age while the risk of thoracic injury and vertebral fractures increased with age (see Table 6).

**Table 6:** Associations between increasing age and ICU, surgery or the risk of specific injuries using logistic regression analysis

	OR	95% CI	p
*ICU*			
Bivariate: increasing age	1.03	1.01–1.04	0.004
Multivariate*: increasing age	1.05	0.99–1.11	0.092
*Surgery*			
Bivariate: increasing age	0.99	0.98–0.99	0.015
Multivariate*: increasing age	0.98	0.96–0.99	0.022
*Upper extremity injury*			
Bivariate: increasing age	0.98	0.97–0.98	< 0.001
Multivariate*: increasing age	0.94	0.93–0.96	< 0.001
*Vertebral fractures*			
Bivariate: increasing age	1.03	1.02–1.04	< 0.001
Multivariate*: increasing age	1.04	1.02–1.07	0.001
*Thoracic injuries*			
Bivariate: increasing age	1.04	1.03–1.06	< 0.001
Multivariate*: increasing age	1.05	1.03–1.08	< 0.001

*OR* odds ratio, *CI* confidence interval, *ICU* intensive care unit, *GCS* Glasgow Coma Scale, *CCI* Charlson Comorbidity Index, *TAL* trauma alert level, *MOI* mechanism of injury, *NOI* number of injuries

*Variables included in model: sex, age, GCS, CCI, TAL, MOI, NOI

## Discussion

This study describes the epidemiology of all equestrian related injuries treated at a Scandinavian trauma centre during a 10-year period. Karolinska University Hospital has a catchment area of over 2.5 million and is the largest trauma centre in Sweden. The aim of this study was to outline the nature of the injuries sustained during equestrian activities, identify outcome trends, and assess the relationship between increasing age and patient outcomes. Our results demonstrate that this group of patients is dominated by the female sex, has a heavy majority of patients who are under the age of 18, and most commonly sustain injuries due to falling off the horse. These findings are corroborated by other studies [3, 15, 16]. Although mortality and the need for intensive care in the total cohort were very low at 0.03% and 1.2%, respectively, 24.9% of the cohort required hospital admission. Out of the group requiring admission, 43.3% needed surgery. Our data show a significant injury burden with 10.7% of patients in the total cohort having an Injury Severity Score (ISS) of nine or higher. This reflects the need for admitting doctors to take this mechanism of injury seriously.

In 2017, the Swedish insurance company Folksam made a compilation over equestrian related injuries reported to them over the course of the year [3]. They demonstrated a national average injury rate of 18 cases per 100,000 population. Our results show an average rate of 14 cases per 100,000 population. The discrepancy between the two may be explained by the fact that this is a single-centre study based on the regional trauma centre only. It is likely that less severe equestrian related injuries were handled by other emergency hospitals in the region and therefore not included in the study cohort. Additionally, the Karolinska trauma centre serves a metropolitan region in which it is likely that fewer people participate in equestrian activities compared to more rural regions and the national average. The report by Folksam placed head and neck injuries on top, constituting 36.4% of all injuries [3]. The result of the current study identifies upper extremity injuries as the most frequent type of injury with a prevalence of 25.6% of all injuries sustained in the total cohort. Amongst upper extremity injury, fractures were by far the most common, making up 17.6% of those injuries. Similar research from other parts of Sweden supports a high frequency in upper extremity injury in the context of equestrian activities [17]. Head and neck injuries do form a close second in our data and constitute 23.9% of all injuries in the total cohort in our study. This slight difference in injury rates between the current study and the compilation of Folksam may be explained by differences in demographics. Folksam’s report consists of national data whereas this study focuses on a single centre which may limit its generalisability. Another possible explanation could be mismatch between actual injuries and the proportion of patients who seek medical attention and claim from their insurance company. Thus, a strength of the current study is the all-encompassing inclusion of patients during a 10-year period without limitation to insurance claims. The injury pattern to the regions of head/neck and extremities found in the current study agrees with other equine trauma publications [18].

The need for intensive care was variable and not significantly associated with age in our data. A total of 36 patients (4.8% of patients admitted to hospital) required ICU admission, 30 out of the 36 had an ISS of nine or higher. The proportion of patients requiring ICU is lower than that of similar studies on equestrian related injury [16]. In the US, more than half of patients admitted to ICU is constituted of older adults [19]. In Hong Kong, general trauma patients have an admission rate to the ICU of 19.8% in the age group 55–70 years and 10.0% in the age group > 70 years [20]. This suggests that there should be a more prominent need for intensive care related to age than what the results of this study indicate. Previous research suggests that even though the mortality of geriatric patients could be reduced by placing appropriate patients in the ICU, geriatric patients are less likely to be triaged to a trauma centre and they, when compared to younger patients with the same ISS, have a lower admission rate to the ICU [21]. Such conflicting findings could be explained by cultural differences and differences in medical traditions that lead to differences in triaging between countries, making comparisons of results between countries challenging.

Furthermore, our data show that equestrian related injuries affect all ages, although more frequently children and adolescents. Our regression models show that the younger patient population requires surgery to a greater extent and more commonly injure their upper extremities, with fractures as the most common injury. One possible explanation is that fractures often require open reduction and internal fixation, which may explain the connection between a young age and the increased need for surgery. An alternate explanation is that conservative treatment, if possible, is more common in the elderly population in contrast to a more operatively aggressive approach in the young. The incidence of vertebral fractures on the other hand increased with increasing age. This type of fracture is often treated non-operatively. The same association was observed with regards to thoracic injuries. The most common thoracic injury in the studied cohort was rib fractures. This type of injury rarely requires surgical intervention and significantly increased with increasing age. Consequently, the association between age and certain types of injuries may offer an explanation to why the prevalence of surgery was slightly lower in the older patient population.

Although there were significant differences found in both injuries and outcomes related to age, the question why remains. One factor could be the dynamics of the accident. Small children are likely to be at a riding school. In this context the focus is on the basics i.e., balance control and communication with the horse. The riding does not entail a lot of risk-taking. When accidents do occur, it is probably due to a surprised child, losing their balance when the pony moves in an unexpected way. To ease the fall, they put their hands out. The impact from a fall like that is still enough to cause a fracture, even if the ride itself is not associated with any greater risks. Adults have better balance and coordination than children. As a group they are also likely to have more experience and their riding would entail higher risks. Their injuries are less likely to be caused by a loss in balance, and more likely to involve a fall at high speed. A fall off the horse in a situation that involves a risk of hitting a fence or a solid object like a tree, offers a situation where hitting the chest or back is more likely. There is a significant divergence in the injury spectrum between different ages. One possible explanation for this could be how the fall occurs. Assumptions that the mechanism of injury is important are strengthened by findings that pinpoint vertebral fractures as more common with increasing age, as our results do, and by associations between certain sports like horse-riding and cycling with an increased risk of vertebral fractures specifically [22]. In martial arts, the wielders are trained to fall in a way that prevents injury. It has been suggested that the same techniques could be used to reduce fall injuries in the geriatric population [23]. One can assume that this would be useful in equestrian activities too. In fact, the SEF has recently commenced recommending their affiliated ring schools to offer training in safe fall techniques as a complement to the riding education.

Equestrian activities are reputed to be dangerous. Nonetheless, during the studied 10-year period there was only one event in the cohort with a deadly outcome. A study conducted in the US with a similar design to the current study showed a mortality rate at approximately 1.3% [24]. This differs from the results presented here. However, the study by Mutore et al. looked at trauma patients only, not covering patients with less severe injuries from the general ED like the current study. Another single centre study performed in Queensland with a study period of 7 years showed a result of three deaths in a population of 171 [25]. Even though the mortality rate in the current study was low, the admission rate was high. Of all patients that sought care at the ED, 24.9% were admitted. This suggests that a significant proportion of equestrian related injuries are severe enough to be taken seriously even if they rarely are deadly. A study conducted at Umeå University Hospital in the north of Sweden, described injuries presented at their ED with focus on patients under the age of 19. They found that the most common mechanism of injury was a fall of some kind. Sports were often associated with injuries and 61% of sports-related injuries were due to football, snowboarding, floorball, ice hockey and equestrian activities [26]. Twelve percent of all patients that sought the ED were admitted. However, differences such as the exclusion of adults and geography must be considered when comparing results.

Furthermore, all-terrain vehicles (ATV) is an activity claimed to be extremely dangerous. A recent review shows high morbidity and mortality. Like injuries sustained from equestrian activities, the most common body part to injure were the extremities and the head. Although the mortality rate from ATV was much higher, the rate of admission was similar to the current study at 30% [27]. In addition, data show that youths involved in ATV trauma were seven times more likely to be hospitalised than the younger paediatric population [28]. A study by Taree et al. compared the rate of surgery amongst 25 competitive sports in the National Collegiate Association (NCAA) [29].When looking at a time period of ten seasons they found 64,598 documented injuries whereof 5.96% required surgery. American football represented most documented injuries and was considered the most dangerous sport. Compared to that study, the current study’s compilation of documented injuries is small. Nonetheless, of all the equestrian related injuries from the admitted subgroup, 43.3% (n = 328) required surgery. Other publications on equine trauma have found similar, or even higher, surgical rates ranging between 24% and 50% [4, 16, 30]. This indicates that injuries sustained in equestrian sports require surgery more frequently than those from many other sports.

The current study has limitations that must be addressed. The lack of exclusion criteria with regards to age must be recognised as a possible source of bias. There is a risk that toddlers who sustained injuries as a result of falling off a horse most likely did so due to underdeveloped motor skills rather than as a consequence of the horse-related activity itself. As such, there is a risk that the prevalence of injuries becomes artificially high. The inclusion of all ages was, however, chosen to be able to answer the primary aim of a full mapping of equestrian related injuries. An important limitation lies in the necessity to retrospectively rely on medical records. This is a weakness of all retrospective studies and needs no further commenting. In the design of a prospective study on the topic of equestrian related injuries the authors recommend the inclusion of data on safety equipment of the rider and knowledge about the rider’s prerequisite at the time of the accident. Information on when and in what context the injury happened, whether it was work-related or not, the degree of rider experience and whether alcohol or drugs were involved would also be of value. For example, Buchanan et al. found an increased risk of injury to the chest and spine in the context of large animal related injuries for riders compared to non-riders [31]. We are unable to assess this finding due to the lack of information about rider experience. Without this kind of information, it is also not possible to make any conclusions about how to avoid certain risks and how to reduce the chance of sustaining certain types of injuries. Another limitation lies in the lack of information on the functional outcome of the patients. This makes it hard to assess the long-term impact of equestrian related injuries on the patient and on society. These limitations can be addressed by a prospective design.

## Conclusions

Of those who seek hospital care due to equestrian related injuries, one in four has injuries considered severe enough to require hospital admission and an overwhelming majority of patients are female. Albeit the morbidity may be high, the mortality is very low. This suggests that equestrian activities are not as deadly as some have assumed. Equestrian activities appear to bare a risk specifically for extremity injury and head and neck trauma, and with increasing age, the risks of thoracic injury and vertebral fractures increase. These findings should be considered when assessing this patient group in the Emergency Department. Increasing knowledge and understanding regarding these injuries can help hospital staff to give better and more efficient care. For those not familiar with horses and the equestrian sports it can be difficult to know what to expect, and what type of history-taking information that could be useful to make the examination and treatment as expedient as possible. We believe that the results outlined in this article can be used to widen this understanding amongst healthcare workers and extrapolated to the creation of diagnostic and therapeutic protocols that may help front-line clinicians in their decision-making.

## Data Availability

Data may be available upon reasonable request if granted by the relevant ethics review board. Author Dr Ahl Hulme can be contacted for this.

## Abbreviations

SEF: Swedish Equestrian Federation
GCS: Glasgow Coma Scale
CCI: Charlson Comorbidity Index
TTAL: Trauma team activation level
MOI: Mechanism of injury
NOI: Number of injured body areas
ISS: Injury Severity Score
SBP: Systolic blood pressure
TBI: Traumatic brain injury
SAH: Subarachnoid haemorrhage
SDH: Subdural haemorrhage
EDH: Epidural haemorrhage
DAI: Diffuse axonal injury
UII: Unspecified intracranial injury
UIH: Unspecified injury to the head
Q1: Lower quartile
Q3: Upper quartile
ICU: Intensive care unit
HLOS: Hospital length of stay
ICU LOS: Intensive care length of stay
OR: Odds ratio
CI: Confidence interval
ATV: All-terrain vehicles
NCA: National Collegiate Association
